# Impact of Mast Cell Activation on Neurodegeneration: A Potential Role for Gut–Brain Axis and *Helicobacter pylori* Infection

**DOI:** 10.3390/neurolint16060127

**Published:** 2024-12-06

**Authors:** Marina Boziki, Paschalis Theotokis, Evangelia Kesidou, Maria Nella, Christos Bakirtzis, Eleni Karafoulidou, Maria Tzitiridou-Chatzopoulou, Michael Doulberis, Evangelos Kazakos, Georgia Deretzi, Nikolaos Grigoriadis, Jannis Kountouras

**Affiliations:** 1 Laboratory of Experimental Neurology and Neuroimmunology, the Multiple Sclerosis Center, 2nd Department of Neurology, AHEPA University Hospital, Aristotle University of Thessaloniki, 54636 Thessaloniki, Greece; ptheotokis@gmail.com (P.T.); bioevangelia@yahoo.gr (E.K.); nellamaria913@gmail.com (M.N.); bakirtzischristos@yahoo.gr (C.B.); elenikarafoulidou95@hotmail.com (E.K.); ngrigoriadis@auth.gr (N.G.); 2 Second Medical Clinic, School of Medicine, Ippokration Hospital, Aristotle University of Thessaloniki, 54642 Thessaloniki, Greece; mtzitiridou@uowm.gr (M.T.-C.); doulberis@gmail.com (M.D.); ekazakos@gmail.com (E.K.); gderetzi@gmail.com (G.D.); 3 Midwifery Department, School of Healthcare Sciences, University of West Macedonia, Koila, 50100 Kozani, Greece; 4 Gastroklinik, Private Gastroenterological Practice, 8810 Horgen, Switzerland; 5 Division of Gastroenterology and Hepatology, Medical University Department, 5001 Aarau, Switzerland; 6 Department of Neurology, Papageorgiou General Hospital, 54629 Thessaloniki, Greece

**Keywords:** mast cells, neurodegeneration, neuroinflammation, Alzheimer’s disease, Parkinson’s disease, amyotrophic lateral sclerosis, multiple sclerosis, gut–brain axis, *Helicobacter pylori*

## Abstract

Background: The innate immune response aims to prevent pathogens from entering the organism and/or to facilitate pathogen clearance. Innate immune cells, such as macrophages, mast cells (MCs), natural killer cells and neutrophils, bear pattern recognition receptors and are thus able to recognize common molecular patterns, such as pathogen-associated molecular patterns (PAMPs), and damage-associated molecular patterns (DAMPs), the later occurring in the context of neuroinflammation. An inflammatory component in the pathology of otherwise “primary cerebrovascular and neurodegenerative” disease has recently been recognized and targeted as a means of therapeutic intervention. Activated MCs are multifunctional effector cells generated from hematopoietic stem cells that, together with dendritic cells, represent first-line immune defense mechanisms against pathogens and/or tissue destruction. Methods: This review aims to summarize evidence of MC implication in the pathogenesis of neurodegenerative diseases, namely, Alzheimer’s disease, Parkinson’s disease, amyotrophic lateral sclerosis, Huntington’s disease, and multiple sclerosis. Results: In view of recent evidence that the gut–brain axis may be implicated in the pathogenesis of neurodegenerative diseases and the characterization of the neuroinflammatory component in the pathology of these diseases, this review also focuses on MCs as potential mediators in the gut–brain axis bi-directional communication and the possible role of *Helicobacter pylori*, a gastric pathogen known to alter the gut–brain axis homeostasis towards local and systemic pro-inflammatory responses. Conclusion: As MCs and *Helicobacter pylori* infection may offer targets of intervention with potential therapeutic implications for neurodegenerative disease, more clinical and translational evidence is needed to elucidate this field.

## 1. Introduction

Neurodegenerative diseases are a heterogenous group of disorders characterized by irreversible neuron loss and the accumulation of progressive neurological disability. What is more, therapeutic approaches utilizing agents with a potential for neuroprotection have, to the most part, failed to tackle disease mechanisms and to ameliorate disease evolution. Although neurodegenerative diseases exhibit heterogeneity regarding clinical presentation, genetic predisposing and/or causative factors, as well as aspects of the underlying pathology, they also share several common characteristics. Cognitive decline, for instance, characterizes Alzheimer’s disease (AD), dementia with Lewy bodies (LBD), Parkinson’s disease (PD) and Huntington’s disease (HD). Motor deficits are prominent in amyotrophic lateral sclerosis (ALS), PD, HD and multiple sclerosis (MS) [1]. Moreover, aging is a major risk factor common in all neurodegenerative diseases [2]. In terms of pathology, neurodegenerative diseases are characterized by the central nervous system (CNS) aggregation of proteins, such as the aggregation of amyloid beta (Aβ) and hyperphosphorylated tau in AD, α-synuclein in PD and in LBD, TAR DNA-binding protein (TDP)-43 in ALS and fronto-temporal dementia (FTD) and Cytosine – Adenine - Guanine (CAG)-polyglutamine protein in HD [3]. The composition of these aggregates is genetically determined, especially in familial cases [4]. Overall, in the context of neurodegenerative disease, the aggregation of these misfolded proteins leads to the dysregulation of several pathways that primarily affect neurons, such as mitochondrial damage, energy defect, production of reactive oxygen species and oxidative stress, synaptic toxicity and, eventually, synapse and neuron loss. The gut–brain axis (GBA) is thought to affect neurodegenerative diseases by several pathways, such as alterations in the immune system via the influence of the gut microbiota, the vagus nerve and the enteric nervous system (ENS), the neuroendocrine system, as well as direct metabolic pathways and hormones [5]. Of note, alterations in the composition of gut commensal microbiota, referred to as “gut dysbiosis” have been reported in patients with AD, PD, ALS and MS, to name few of the neurodegenerative diseases [6,7,8]. Experimental studies also indicate pathways by which the GBA affects the pathogenesis of neurodegenerative diseases, such as CNS innate immune system alterations, microglial activation and propagation of neuroinflammatory responses [9,10].

Recently, a large body of evidence supports neuroinflammation as a crucial component of the pathology that underlies the otherwise regarded as “primary neurodegenerative disease such as AD, PD, ALS and HD. Conversely, for MS, traditionally regarded as a disease of neuroinflammatory origin, with secondary neurodegeneration occurring later in the disease’s course, it is nowadays widely acknowledged that neurodegeneration is also present at the initial disease stages, to a lesser degree, compared to the later stages. These observations suggest that neuroinflammation and neurodegeneration are both crucial players in these pathological processes. Moreover, due to the lack of successful treatments for neurodegenerative diseases so far, modulation of the immune responses involved in neurodegeneration appears to be a promising therapeutic strategy, further supported by evidence stemming from experimental and clinical studies. Several of these approaches attempt to modulate signaling pathways and cellular components of the innate immune system. Mast cells (MCs) are multifunctional effector cells that, together with dendritic cells, represent first-line immune defense mechanisms against pathogens and/or tissue destruction. When MCs are activated, depending on either physiological and/or pathological conditions, they degranulate and release several mediating molecules. For instance, MCs participate in the regulation of vasodilation and the overall vascular homeostasis, and they are bearing potential to trigger responses of the innate and adaptive immune system. Moreover, MCs are cellular mediators of several pathologies, such as allergic reactions, asthma, gastrointestinal (GI) diseases, cancer and cardiovascular disease (CVD). MCs are normally located in mucosal and epithelial tissues, as well as in other vascularized tissues, with the possible exception of the CNS and the retina [11]; in normal human brains, MCs are not numerous [12]. MCs are cellular components of the innate immune system and, as such, they are abundant at sites of the body where the host and the external environment interact, as well as at sites that often serve as entry points for antigens (the GI tract, the skin and/or the respiratory epithelium) [13]. Human MCs exhibit two phenotypes: the mucosal MCs, producing tryptase, and the connective tissue MCs, producing additional mediators, such as chymase and carboxypeptidases [14,15]. In this respect, MCs are a component of the innate immune system that may bear potential as a possible target of therapeutic intervention for disease. This review aims to summarize evidence of MC implication in the pathogenesis of neurodegenerative diseases, namely, AD, PD, ALS, HD and MS. In view of recent evidence that the GBA may be implicated the pathogenesis of neurodegenerative diseases, this review also focuses on the MCs as potential mediators in the GBA bi-directional communication and the possible role of *Helicobacter pylori* (*Hp*), a gastric pathogen known to alter the GBA homeostasis towards local and systemic pro-inflammatory responses. As MCs and *Hp* infection may offer targets of intervention with potential therapeutic implications for neurodegenerative diseases, more clinical and translational evidence is needed to elucidate in depth this field.

## 2. Methodology

Regarding the strategy of sources and methods used, an online literature search was conducted in PubMed, EMBASE and Web of science databases, without time restrictions. Search phrases included the keywords or MESH (mast cells and central nervous system or neurodegenerative disease or neuroinflammation or intestine or immune system or Alzheimer’s disease or Parkinson’s disease or Amyotrophic Lateral Sclerosis or Huntington’s disease or Multiple Sclerosis, mast cells and intestine or gut–brain axis or *Helicobacter pylori*). Research and clinical studies in humans, animals, tissues and cell cultures were included. Original articles, patient series and reviews, systematic and meta-analyses and book chapters were retrieved. The literature list of the reviews was manually searched and additional studies that met the selection criteria of this review were selected. With respect to the study selection criteria, although no limitation regarding the year of publication was used in the search, studies and articles from the last five years were preferred, with a particular focus on clinical and laboratory experimental studies. However, several older studies were also included, for reasons of completion. Articles written or translated into English were included. Reviews were also considered appropriate for inclusion as sources for cited studies that were not retrieved in the initial literature search with the respective keywords.

## 3. Neuroinflammation and Neurodegenerative Diseases

Inflammatory response may be either acute, developing from seconds to hours and frequently acting as a beneficial mechanism, or chronic, with a longer duration that may cause tissue destruction. Chronic inflammation develops either due to the failing of the several inflammatory response’s regulatory mechanisms, or in the presence of over-activation of the immune system [16,17]. The innate immune response aims at preventing pathogens from entering the organism and/or to facilitate pathogen clearance via a non-specific mechanism in terms of antigen recognition. Non-specific defense mechanisms towards pathogens include physical and chemical barriers. Innate immune cells, such as macrophages, MCs, natural killer (NK) cells and neutrophils bear pattern recognition receptors (PRRs) and are thus able to recognize molecular patterns, common across a wide variety of pathogens, namely, pathogen-associated molecular patterns (PAMPs), as well as molecules derived from tissue destruction, namely, damage-associated molecular patterns (DAMPs) as it occurs in the context of so-called neuroinflammation [18]. 

Inflammation in the nervous system, termed as aforementioned “neuroinflammation”, can be particularly detrimental [17]. Microglia are resident cells of the innate immune system of the CNS. Upon chronic inflammation microglia secretes pro-inflammatory cytokines that exert tissue detrimental effect, whereas, upon the initial immune response, its activation is considered mostly beneficial [17,19,20,21,22]. Microglia, scavenger cells of the CNS, MCs and astrocytes comprise the main sources in the CNS milieu of pro-inflammatory cytokines [17,23]. Chronic disorders, such as cerebrovascular disease, type 2 diabetes mellitus (T2DM) and rheumatoid arthritis, are subjected to the effect of systemic inflammation [19]. Moreover, the Western diet and overall lifestyle is linked to obesity, insulin resistance (IR), hypercholesterolemia and metabolic (dysfunction) associated steatotic disease (MASLD), [24,25,26] contributing factors to metabolic syndrome (MetS), a common denominator for several CNS pathologies, such as cerebrovascular and neurodegenerative diseases [27]. An inflammatory component in the pathology of otherwise “primary cerebrovascular and neurodegenerative” disease has recently been recognized and targeted as a means of therapeutic intervention [28]. 

## 4. Mast Cells (MCs)

Activated MCs are multifunctional effector cells generated from hematopoietic stem cells that, together with dendritic cells, represent first-line immune defense mechanisms against pathogens [29,30]. MCs differentiate in the bone marrow in the presence of c-kit ligand and interleukin 3 (IL-3) [31] and are subsequently inserted in the blood circulation [29,30]. Their maturation occurs following to their migration to various tissues and organs in the presence of the local tissue microenvironment [32,33]. MCs are prevalent in host/environment interfaces such as the skin, lung tissue, GI and urogenital tracts, as well as the connective tissue adjacent to blood and lymphatic vessels. Conversely, they are absent from avascular tissues (cornea, mineralized bone and cartilage) [11]. Furthermore, MCs circulate from the periphery towards the CNS upon neonatal development and in the adult [34,35,36]. MCs are also found in small numbers in the healthy brain, in perivascular areas in the meninges [37] and other parts of the CNS (i.e., dorsal medulla, choroid plexus, thalami and hypothalamus) [38,39]. Upon injury, illness or stress, additional MCs are recruited in the CNS [40,41,42]. In the spinal cord, MCs are mainly distributed in the dura, where they secrete immune mediating molecules that regulate synaptic transmission in the proximal CNS regions [43,44]. MCs are also prevalent in several tissues, in close proximity to peripheral nerves [45,46,47,48]. 

MCs are thought to be implicated in physiological homeostatic processes and immune defense mechanisms with immediate and/or delayed effects [49,50]. Specifically, MCs can rapidly, within minutes, secrete cytokines, leukotrienes and other inflammatory mediators in response to allergens, antigens, complement factors, neuropeptides, medications, and trauma [29,34]. Moreover, MCs bear the ability to interact with surrounding cells and structures, such as arteries and nerve fibers, via the secretion of extracellular vesicles, traps and nanotubes [46,51]. MCs in the CNS exhibit the potential of antigen presentation, pro-inflammatory molecule release and phagocytosis. Lastly, the degranulation of MCs has been shown to increase neuronal excitotoxicity [52] and to enhance the homeostatic responses to trauma and stress [53,54]. 

## 5. Mast Cells (MCs) and the Gut–Brain Axis (GBA)

**Figure 1:**
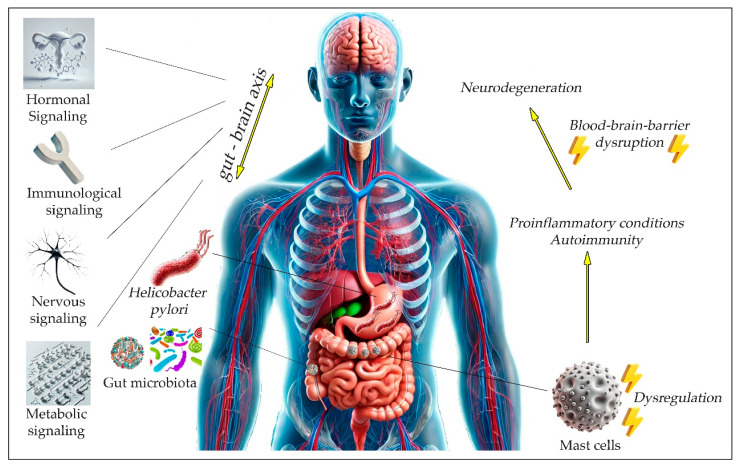
The gut–brain axis (GBA) as a bidirectional network. The figure illustrates the gut–brain axis as a bidirectional network controlled by hormonal, nervous, immunological, and metabolic signaling pathways. It shows how dysregulated mast cells disrupt the gut microbiome, leading to inflammation and gut barrier breakdown. The role of *Helicobacter pylori* is highlighted, showing its contribution to neurodegenerative diseases through immune mechanisms, including defective monocytes and molecular mimicry, which may lead to neuroinflammation and blood–brain barrier disruption. single-headed yellow arrows denote result; double-headed yellow arrows denote bi-directional interaction/communication; yellow lightning signs denote pathology.

The GBA is a complex network that describes the bilateral interaction between the CNS and the gut, including the ENS, through various mechanisms that consist of nervous, hormonal, metabolic and immunological signaling [55]. Consequently, the GBA plays a pivotal role in regulating multiple functions such as the CNS function, emotional regulation, neuromuscular regulation and immunity. Current research increasingly focuses on unveiling the connection between CNS higher functions and GI processes, including gut microbiota symbiosis [55,56]. Because of the established function of the gut microbiome in the maintenance of a physiological brain–gut interaction and its participation in the pathogenesis of various disorders, this relationship has recently been termed the “microbiota—gut—brain axis” (Figure 1) [57].

Gut microbiota consists of more than 100 trillion microorganisms that inhabit the human digestive tract [58]; their number is approximately 10 times that of total human cells, with above 1000 diverse species [59]. Not only do the gut microbiota serve as a natural protective barrier against pathogens, but they also participate in the regulation of the neurotransmission and in neuromodulation pathways via the regulation, at least in part, of the biosynthesis as well as the production of serotonin, aminobutyric acid, dopamine and short chain fatty acids (SCFAs) [60]. The intestinal epithelial barrier is a key player in maintaining tissue homeostasis through immune responses which are coordinated via internal and external signals. MCs are abundant in the intestinal mucosa, where they pose both defense- and immune-regulatory role. MC dysregulation may disrupt GI and gut microbiota homeostasis, which induces pro-inflammatory conditions and/or autoimmunity, locally in the intestinal mucosa [61]. Increasing evidence recently established the critical importance of the interplay among gut microbiota, CNS and MCs in neurodegenerative disorders [62]. The rising incidence of neurodegenerative disorders may be promoted through CNS–MCs triggering leading to blood–brain barrier (BBB) disruption, neuro-immune activation and synaptic dysfunction. In the case of neuroinflammatory conditions, chemokines and other immune mediators are produced and migrate through the GBA causing intestinal MCs degranulation which subsequently leads to gut-barrier breakdown. 

## 6. *Helicobacter pylori* and Neurodegenerative Diseases

*Helicobacter pylori* (*Hp*) is a Gram-negative, microaerophilic spiral-shaped and flagellated [63] bacterium, prevalent in about 58% of the world’s population [64], being implicated in the pathogenesis of duodenal and gastric ulcer disease, as well as of gastric malignancies [65]. The main natural permanent reservoirs for *Hp* include the gastric epithelium and the oral cavity [66,67,68]. The gut appears to be a secondary location for *Hp* [69,70]. When left without treatment, Hp may cause a chronic infection and histological inflammation (gastritis) that can endure for many years, if not throughout lifetime. *Hp* is one of the most widespread human infectious agents [71,72,73], and affects above 4.4 billion individuals worldwide [74]. *Hp* infection is facilitated by genetic diversity, as well as the effective evasion of the host innate and adaptive immune response. *Hp* genetic diversity results from a high mutation rate, which is aided by the absence of a DNA mismatch repair mechanism and the acquisition of DNA from other strains [75]. By eliminating phosphate groups from the lipid A, the *Hp* lipopolysaccharide evades recognition by antimicrobial peptides and Toll-like receptors (TLRs) [76]. Vacuolating cytotoxin A (VacA), which is secreted by *Hp*, inhibits nuclear translocation of the nuclear factor of activated T-cells (NF-AT), which regulates T cell proliferation [77]. Beyond its impact on the GI tract, many investigations have implicated *Hp* infection in a variety of diseases, including neurological and neurodegenerative disease [65,78,79,80,81].

### 6.1. Alzheimer’s Disease (AD)

A link between *Hp* infection and AD has previously been described [82,83,84,85,86]. We previously [87] hypothesized that *Hp* acts through immune-mediated mechanisms according to which secreted interleukins, interferons and other inflammatory mediators orchestrate the effect of the acute and chronic phase of the inflammatory response towards *Hp*. In this respect, mononuclear cells may be stimulated to create a tissue factor-like procoagulant which converts fibrinogen to fibrin. A detailed review of the current findings on humoral and cellular immune dysregulation elucidated a potential relationship between *Hp*-infection and neurodegenerative conditions, including AD [85,87]. We and others also proposed that cognitive impairment may be precipitated by *Hp* infection in the context of anti-*Hp* IgG that targets the brain milieu [87,88]. Furthermore, experimental data indicate the abundance of IL-1 pro-inflammatory cytokine, secreted by microglia and astrocytes, in amyloid plaques, as a common trait in human AD brains as well as in animal models of AD [89,90,91]. IL-1 synthesis is based on MAPK activation and the NF-κB signaling cascade and overexpressed IL-1 enhances phosphorylation of tau protein and the development of neurofibrillary tangles due to GSK-3 activation [92]. The B subunit of *Hp*-Urease (HPU) activates the NOD-like receptor protein 3 (NLRP3) inflammasome, a mechanism upstream of the IL-1 production, by a TLR2-dependent process [93,94].

*Hp* infection appears to be involved in AD pathophysiology by several additional mechanisms. For instance, *Hp* infection may be associated with the brain pericyte dysfunction [95], which could contribute to the pathophysiology of neurodegenerative disorders, such as AD [96]. Likewise, *Hp* infection-related hyperhomocysteinemia appears to contribute to atherosclerosis, which is connected with systemic disorders, including cardio-cerebrovascular and neurodegenerative diseases, such as AD [86]. Hyperhomocysteinemia is an active player implicated in the pathophysiology of mild cognitive impairment, a prodromal condition strongly predictive of subsequent AD development [80], and AD sequence by provoking Aβ and tau pathologies, along with synaptic dysfunction, neuroinflammatory process and memory decline. This suggests a new therapeutic strategy for patients exhibiting this risk factor [97]. Related studies provide evidence of the connection between *Hp* infection and AD-like Aβ and phospho-tau pathology, signifying that *Hp* eradication may offer benefits in the prevention of tauopathy [98]. Moreover, *Hp* infection is associated with atrial fibrillation (AF), *Hp* is an independent predictor for long-lasting AF [99], and AF is closely linked to AD and cognitive decline [100]. Beyond catheter ablation for AF management [100], *Hp* eradication may also reduce the risk of AF-related AD, and further investigation is required. Additionally, *Hp* is related to galectin-3, which is significantly associated with the severity of memory loss and AD stage [84]. Galectin-3 inhibitors appear to suppress microglial activation [101], suggesting an exciting therapeutic target to impede neurodegenerative disorders, including AD. Furthermore, *Hp*, by promoting gut dysbiosis, might lead to the brain disorders such as AD [102]. Gut dysbiosis plays an important role in the pathophysiology of AD, by influencing neuroinflammation and disease progression. Related therapeutic approaches, including probiotics, prebiotics and fecal microbiota transplantation, offer benefits in AD pathology [103]. *Hp* eradication may also positively influence AD manifestations at 2- and 5-year clinical endpoints, thereby contributing to the long-term survival rate of this disorder [104].

### 6.2. Parkinson’s Disease (PD)

Increasing evidence indicates higher prevalence of *Hp* infection in people with PD, compared to the general population, though, the exact cause for this association remains to be elucidated. *Hp* toxins, gut dysbiosis [105], excess of pro-inflammatory molecules [106,107], *Hp* antigens and molecular mimicry towards the host’s neuronal proteins [108] as well as a possible effect of *Hp* on levodopa pharmacokinetics [109], are all advocated as possible pathogenic mechanisms. Moreover, the emergence of motor symptoms in the context of PD may be precipitated by mechanisms acting on the bidirectional micobiome—GBA [110,111]. For instance, patients with PD exhibit lower levels of gut beneficial fecal of SCFA, as well as lower relative abundance of SCFA-producing bacteria, compared to healthy controls [112]. Lack of bacteria-produced SCFA, namely, butyrate, has also been implicated in the progression of PD [113]. Ιn a single-center, double-blinded, randomized placebo-controlled study, *Hp* eradication did not improve clinical outcomes in PD, suggesting no potential benefit from routine *Hp*-screening or *Hp*-eradication in the management of PD [114]. Nevertheless, *Hp* eradication in patients with PD might improve the bioavailability of L-3,4-dihydroxyphenylalanine (L-dopa), a precursor of dopamine used as a treatment for PD, and reduce motor fluctuations [115]. Moreover, *Hp*, which colonizes predominantly the gastric epithelium and oral cavity, is a risk factor for periodontitis [116], which, beyond AD [117], is also linked to PD [118]. *Hp* eradication and treatment of periodontitis are beneficial for the prevention of PD and dementia [118]. Thus, further related prospective studies are warranted to clarify this critical issue.

### 6.3. Amyotrophic Lateral Sclerosis (ALS)

*Helicobacter* spp., together with *Pasteurella pneumotropica*, *Tritrichomonas muris* and murine norovirus were significantly more abundant in C9orf72Harvard mice than in C9orf72Broad mice [10]. This increase in *Helicobacter* spp. relative abundance suggested a possible link with increased mortality and inflammation in the C9orf72Harvard mice, whereas administration of broad-spectrum antibiotics lead to a reduction in inflammation and improved clinical outcomes. To our knowledge, however, no clinical evidence of an association between *Hp* and/or other *Helicobacter* species with ALS in humans has been reported.

### 6.4. Huntington’s Disease (HD)

In a retrospective study, the results of 105 esophagogastroduodenoscopies, performed in 68 patients with HD, showed that *Hp* was positive in 4 of 19 patients investigated (21.1%). A high prevalence of gastritis or esophagitis was reported [119]. Although this finding indicates an association of HD with dysregulation of the GI system, thus posing potential implication for the role of the GBA in HD, this assumption warrants for a more detailed investigation. To our knowledge, however, additional clinical and/or experimental evidence regarding potential association between *Hp* with HD is currently lacking.

### 6.5. Multiple Sclerosis (MS)

While a robust link between *Hp*-infection and MS was not demonstrated in initial several observational studies, a noteworthy occurrence of concomitant autoimmune disorders among MS patients was indicated [78]. Patients with MS also exhibited atrophic gastritis evidenced by histology. Molecular mimicry is an intriguing assumption for this association: *Hp*-infection may induce immune responses following to an erroneous cross-reaction of *Hp* antigens with antigens of the peripheral and the CNS, thus potentially initiating and perpetuating nervous tissue damage [78]. Another study identified galectin-3 as a potential antigen recognized by antibodies present in the peripheral blood of individuals with secondary progressive MS (SPMS) [120]. Although these antibodies were advocated to serve as potential novel marker for SPMS diagnosis and as a potential target for therapeutic agents [120], the exact implication that these antibodies pose for SPMS remains to be elucidated. Galectins, being highly conserved molecules across a wide range of epithelial and immune cells, may explain some of the phenotypic and functional variation in the otherwise well-characterized TLR-dependent signaling, induced by several microbes and/or pathogens, including *Hp* [121]. Specifically, galectin-3 increased expression is reported in active injured regions in MS patients [122], and in postmortem MS human brain tissues [123], thereby appearing to be an important therapeutic target in neurodegenerative disorders including MS [124]. Greek and other data indicate that active *Hp* infection, apart from MS [78,125,126], is also common in patients with clinically isolated syndrome (CIS), the prodromal phase of consequent development of MS, accompanied by the mentioned hyperhomocysteinemia [79]. *Hp* eradication could delay the progression of CIS to the development of MS [79].

Finally, *Hp*-induced vacuolating cytotoxin A (VacA), displays chemotactic activities toward bone marrow-derived MCs (BMD-MCs) and induces them to produce pro-inflammatory cytokines that, in addition to causing local pathologies [127], lead to disruptions of the blood–brain barrier (BBB) and the blood-ocular barrier (BOB), resulting in neuropathies such as MS, AD and glaucoma, the latter referred to as ‘ocular AD’ [128]. Likewise, *Hp*-related VacA appears to promote the intracellular survival of *Hp*, and activated monocytes (possibly infected with *Hp* due to defective autophagy) could access the brain (Trojan horse theory) through BBB/BOB disruption, thereby triggering the development and progression of neurodegenerative disorders [129], possibly by inducing additional defensin abnormal expression. When *Hp* accesses the brain, it may trigger the maturation and activation of defensin-related dendritic cells leading to the release of pro-inflammatory cytokine by effector T cells, thereby promoting neuronal cell injury and death [130]. Interestingly, defensin-related activation of MCs [131] may also trigger neurogenic disorders, and thus further research is needed.

## 7. Mast Cells (MCs) and Mechanisms of Neuroinflammation in Neurodegenerative Diseases

Evidence of neuroinflammation in the context of neurodegenerative disease has been the focus of extensive research and MCs, as a cellular component of the innate immune system, have been shown to participate inflammatory responses in the CNS in several neurodegenerative diseases (Table 1)

### 7.1. Mast Cells (MCs) in Alzheimer’s Disease (AD)

AD is a prevalent neurological disorder [174,175]. Memory loss is a common symptom of the disease, but there is also a considerable reduction in other cognitive domains (language, visual–spatial skills, praxis skills, reasoning and judgment) [176]. It is estimated that more than 100 million individuals will have dementia by 2050, placing a significant economic impact on societies and families [177]. The main AD pathophysiological characteristics in the CNS are Aβ accumulation, formation of neurofibrillary tangles and abnormal phosphorylation of tau protein, resulting in impaired synaptic function and cognition [178,179]. The primary basis of the amyloid hypothesis of AD is based on the notion that AD pathology is characterized by the accumulation of Aβ in brain senile plaques. However, the prevailing viewpoint for AD has evolved, for AD to be considered as a condition in which soluble oligomeric Aβ forms play a crucial role in disrupting synaptic plasticity and cognition [17]. Although in the normal aging brain, evidence of limited neuroinflammation exists, the critical role of neuroinflammation in the onset and progression of AD, based on the abundance of inflammatory cytokines and microglia in amyloid plaques has long been suspected [138]. Increased exposure to continuous stimuli produces a phenotypic transformation in microglia into a dysfunctional senescent state with limited capacity for phagocytosis and immune tolerance, thus further promoting brain tissue pathology [180].

Neuroinflammation enhances the pathology of AD via several mechanisms including oxidative stress [132], hyperphosphorylation of tau protein [136], Aβ buildup [138] and the impairment of cholinergic transmission [139]. Neuroinflammatory key players such as IL-1, IL-6, and IFN-γ recruit macrophages and other immune cells, such as MCs, into the amyloid plaques, thus further contributing to AD pathogenesis [133,134,135]. Conversely, phagocyted amyloid particles inside MCs were found in skin and gastric samples, where they may trigger histamine release [137]. In addition to Aβ fragments, the amyloid A protein precursor serum amyloid A (SAA), is increased in AD brains [140]. SAA causes MCs degranulation, cytokine release, especially the pro-inflammatory tumor necrosis factor alpha (TNF-α) and IL-1, and chemotaxis [137]. MC granule material causes SAA degradation into proto-fibrillar intermediates [140]. Furthermore, exposure to amyloid peptide fragment 25–35 causes histamine release by BMD-MCs, via a mechanism involving pannexin 1 (panx1) hemichannel (HC) [135]. Panx1 organizes a membrane channel that serves as a conduit for ATP release extracellularly [137]. This channel’s activity can be enhanced with an increase in intracellular [Ca2+], extracellular [K+], and alkaline pH, whereas protein kinase A activation reduces the activity of this channel [135,181]. Additionally, it has been noted that Aβ also contributes to the degranulation of prefrontal cortical MCs, via the enhancement of connexin-43 (Cx43) and Panx1 HC flux [137]. MC abundance in the brains of amyloid precursor protein (APP) and presenilin 1 (PS1) APPswe/PS1dE9 mice significantly surpassed MC abundance in control mice, more notably in the hippocampus and the cortex [137]. Moreover, in APPswe/PS1dE9 mice, prefrontal cortex MCs exhibited basal ethidium bromide uptake, which was reduced significantly by Panx1 and Cx43 HC inhibitors, further suggesting concurrent involvement of these channels in MC activation [137]. 

During the evolution of AD, especially prior to the amyloid plaque formation, MCs are reported to detect low-solubility amyloid particles and to migrate to plaque sites, where they trigger the release of inflammatory mediators and disrupt the BBB [137]. Upon activation, MCs release protease gelatinases (metalloproteinases 2 and 9) and vascular endothelial growth factor (VEGF), leading to vascular leakage, CNS leukocyte infiltration and edema [141]. Upon the development of amyloid plaques, reactive microglia release pro-inflammatory cytokines and glutamate/ATP through HCs, possibly continuously activating Cx43 HCs in nearby microglia and astrocytes [182]. Also in other pathologies with inflammatory components, such as cerebral ischemia [141], traumatic brain injury (TBI) [183], experimental autoimmune encephalomyelitis (EAE) [50] and stress [184], MC activation was shown to exacerbate BBB permeabilization. 

MCs can also detect IL-33, a cytokine released by damaged cells. Moreover, MCs reciprocally induce glia and neurons to further produce IL-33, thus further activating MCs [142,146]. These MCs act as early responders to brain injury, as they release pre-stored TNF and proteases and recruit immune cells, such as neutrophils, within hours form the initial injury [142]. TNF, released by MCs, upregulates adhesion molecules in vascular endothelial cells, thus promoting leukocyte recruitment [143,144]. VEGFs released at injury sites stimulate MC accumulation through VEGF receptors (VEGFR) [145]. Conversely, transforming growth factor beta (TGF-β) derived from MC granules suppresses MC activity and bears role in fibrosis, angiogenesis, tissue repair and the inhibition of immune response. As a response to traumatic brain injury (TBI), MCs were shown to infiltrate the brain tissue and to degranulate, thus releasing pre-stored neuroprotective mediators [185]. However, in severe injuries, the innate immune response leads to chronic neuroinflammation, continuous production of pro-inflammatory mediators, sustained tissue damage and neurobehavioral abnormalities associated with neuronal loss. In this respect, TBI and stress have been advocated as factors to increase AD risk in susceptible individuals, accelerating AD onset and progression. While some studies report no direct link between TBI and AD, the majority support such association [142].

Chronic stress may also accelerate AD progression by the promotion of inflammation and the enhancement of Aβ accumulation, tau hyperphosphorylation, oxidative stress, mitochondrial dysfunction and glucose metabolism dysregulation [186]. In this respect, MCs play a critical role in stress-induced inflammation. MCs release the corticotropin-releasing hormone (CRH), which further activates neighboring MCs and microglia [147]. Chronic stress triggers MC degranulation via the hypothalamic–pituitary–adrenal (HPA) axis, causing excessive pro-inflammatory mediator release, synaptic loss, BBB disruption and neuroinflammation in affected brain regions [146]. Evidence suggests that MC-derived inflammatory mediators may hasten AD progression [187]. 

### 7.2. Mast Cells (MCs) in Parkinson’s Disease (PD)

PD, the second most prevalent age-related neurodegenerative disorder globally, is characterized clinically by typical motor symptoms, including resting tremor, bradykinesia, rigidity, as well as non-motor symptoms [149]. Key pathological features of PD encompass the loss of dopaminergic neurons in the substantia nigra (SN) and ventral tegmental area (VTA), leading to reduced dopaminergic neurotransmission to the striatum (STR), and the formation of α-synuclein bodies. Severe motor deterioration typically manifests in the middle to late stages of the disease [148]. However, the precise pathogenic mechanisms responsible for PD remain elusive. Emerging evidence also links PD progression to intracellular mitochondrial dysfunction-induced reactive oxygen species generation, neuroinflammation, dysfunction in the ubiquitin–proteasomal and autophagy–lysosomal systems, as well as the accumulation of α-synuclein [152]. 

Neuroinflammation has been reportedly linked with all aspects of PD pathology, from α-synuclein aggregation to dopaminergic cell loss and the development of PD symptoms. Activated microglia and astrocytes that accompany midbrain dopaminergic neuron loss are the hallmarks of neuroinflammation in patients with PD and in PD animal models [149]. PD-associated factors, including key gene mutations [α-synuclein (SNCA), Parkin, protein deglycase DJ-1, Leucine-rich repeat kinase 2 (LRRK2)] and neurotoxins (rotenone, methyl-4-phenyl-1,2,3,6-tetrahydropyridine) can contribute in microglia and astrocyte activation [149]. Neuroinflammation plays pivotal role in PD pathogenic processes, contributing to and aggravated by protein aggregation, oxidative stress, mitochondrial dysfunction, calcium homeostasis disruption, and iron deposition [188]. Accumulation of misfolded α-synuclein is linked to dysregulation of immune response in the CNS [151]. Moreover, genome-wide association studies support the neuroinflammatory hypothesis for sporadic PD, identifying PD-associated polymorphisms in the human leukocyte antigen (HLA) region, particularly the HLA-DR gene [151]. Lastly, processes such as neurotoxic substance release by reactive microglia and astrocytes, brain infiltration and activation of inflammatory MCs and T lymphocytes, increased oxidative stress, and increased expression of inflammatory signaling molecules were shown to contribute to PD-related neuronal death and neurodegeneration [151,189].

In neuroinflammatory conditions, glial cells, neurons and MCs communicate via various signaling and mediating molecules [CD40 ligand (CD40L), CD40, TLR2, TLR4, protease-activated receptor-2 (PAR2), chemokine C-X-C receptors 4/12 (CXCR4/CXCL12), C5a receptor], promoting glial activation and further inflammatory mediator release [150]. MCs are advocated as pivotal intermediaries that bridge neurons and neuroinflammation [150]. Specifically, CD40-CD40L interaction triggers pro-inflammatory mediator production; 1-methyl-4-phenylpyridinium (MPP+), a metabolite of neurotoxin 1-methyl-4-phenyl-1,2,3,6-tetrahydropyridine (MPTP) elevates CD40L expression in BMD-MCs, highlighting MCs’ role in neuroinflammation [150]. In the context of PD, brain MCs exert neurotoxic effects, thus contributing to neurodegeneration particularly in the SN [150]. Of note, administration of MPTP after MC reconstitution was shown to increase oxidative stress and altered levels of malondialdehyde, Glutathione, Superoxide dismutase, and Glutathione peroxidase [152]. Lastly, the upregulation of calpain 1 and intercellular Adhesion Molecule 1 (ICAM 1) expression in response to MPTP after MC reconstitution was shown to result into astroglia activation-dependent dopaminergic cell death and motor behavior deterioration in vivo, highlighting the potential therapeutic benefit of GMF inhibition in the context of neuroinflammation that accompanies neurodegeneration [152].

These results are further supported by the report that MCs are attracted and activated in the SN region of MPTP-induced mice through C-C motif chemokine ligand 2/C-C chemokine receptor type 2 (CCL2/CCR2) up-regulation and the expression of adhesion molecules [vascular cell adhesion molecule 1 (VCAM-1), platelet endothelial cell adhesion molecule-1 (PECAM-1), and ICAM-1) [153]. NF-κB activation induces transglutaminase 2 (TG2) expression in activated MCs, releasing inflammatory mediators like histamine, leukotrienes, TNF-α, and IL-1, potentially contributing to tyrosine hydroxylase (TH) + dopaminergic (DA) neuron loss in the SN region. While a similar process may occur in human PD patients, more research is needed to confirm these experimental results [153]. Moreover, MC proteases, namely mouse mast cell protease 6 (MMCP-6) and mouse mast cell protease 7 (MMCP-7), were also shown to stimulate astrocytes and neurons [146]. More specifically, MPP+ triggers MCs to release IL-33 by activating extracellular signal-regulated kinase ½ (ERK1/2), p38 mitogen-activated protein kinase (MAPKs), and NF-κB. Furthermore, in PD brains, particularly in the midbrain and striatum regions, there is an increase in MC numbers, activation status, and IL-33 expression linked to cell injury. The latter suggests that targeting glia-neuron and MC activation pathways may represent a novel therapeutic approach for PD [146].

The NLRP3 inflammasome’s significant involvement in PD has recently come to light. Cleaved caspase-1, IL-1, NLRP3 and apoptosis-associated speck-like protein containing a caspase recruitment domain (ASC)-specks were detected in postmortem PD brain tissues and plasma from PD patients, indicating an overall increase in inflammasome activity compared to age-matched controls [132,154,155]. Moreover, heightened systemic NLRP3 activation has been linked to declining motor function and PD progression [190]. In experimental disease, particularly the MPTP mouse model, a strong association between α-synuclein levels, microglial NLRP3 inflammasome activation and dopaminergic neuron toxicity has been shown [132]. While CNS MC expression of the NLRP3 inflammasome has not been directly studied, peripheral tissue MCs in cryopyrin-associated periodic syndrome patients evidently express functional inflammasomes and produce IL-1 [156]. With respect to the CNS MCs, MC inflammasome was shown to play a pivotal role in the meningeal inflammation that, at least in part, determines disease severity in a rodent model of MS [171]. Taken together, these findings emphasize the central role of MCs as primary instigators of inflammatory responses and suggest that, although direct evidence of NLRP3 inflammasome involvement in brain MC activation and function in neurodegenerative diseases, such as PD and AD is currently lacking, a potential role for CNS MCs in the pathogenesis of these diseases is reasonable to be hypothesized and further investigated. 

Chronic stress can trigger the HPA axis activation, thus potentially contributing to the loss of dopaminergic neurons in the SN in PD [191]. The expression of corticotropin-releasing hormone receptor 1 (CRH-R1) receptors in dopaminergic neurons, depending on their brain location, may exert anxiolytic effects [192]. Microglia and astrocytes surround MCs and can become activated in PD, releasing inflammatory cytokines, CRH, neurotensin, and neuromodulators that stimulate bidirectional communication capable of damaging dopaminergic neurons [150]. Further research is needed in order to better understand the molecular and cellular communication pathways that exist between MCs, CRH neurons and glial cells in the etiology of PD [178].

### 7.3. Mast Cells (MCs) in Amyotrophic Lateral Sclerosis (ALS)

ALS is a heterogenous disease with genetic predisposition, also thought to have multigenetic and possibly environmental causes [159]. Autosomal dominant mutations have been primarily, but not exclusively, related to ALS, namely, the C9orf72, superoxide dismutase 1 (SOD1), transactive response DNA binding protein (TARDBP) and fused in sarcoma/translocated in liposarcoma (FUS/TLS) mutation, among others [193]. ALS diagnosis is challenging, particularly at early stages. Typically, more common diseases are initially investigated, thus resulting in significant diagnostic delay [194]. The pathophysiological hallmarks of ALS are degeneration of motor neurons and distal motor axonopathy. Motor axon degeneration in the context of ALS has been linked to impaired axonal transport and mitochondrial function defect [157]. 

Reactive immune cells have been identified in postmortem tissue of patients with familial and sporadic ALS. More specifically, degenerating motor neuron cell bodies and neuronal axons were reportedly surrounded by microglial cells and/or CNS macrophages. Neuroinflammation has been described as a pathogenic process in ALS. For instance, degranulating MCs have been reported in the quadriceps muscle of patients with ALS but not in controls. In this context, MCs were shown to interact with myofibers and motor endplates. MCs and neutrophils are reportedly surrounding motor axons in SOD1G93A rats with ALS, thus indicating that infiltration by immune cells in ALS extends across the peripheral motor pathway. Moreover, administration of masitinib, a tyrosine kinase inhibitor, resulted in reduced infiltration by MCs and neutrophils, ameliorated axonal pathology and secondary demyelination, and hindered the loss of type 2B myofibers in SOD1G93A rats [158,159]. The tyrosine kinase receptor c-Kit (KIT or CD117) is a member of the transmembrane growth factor receptor family [164], also known as MC growth factor [164]. MCs express c-Kit throughout their lifetime [164] and the stem cell factor (SCF) /c-Kit pathway is essential for MCs in order to survive and to differentiate [164]. Moreover, it has been shown to be necessary in order for MCs to infiltrate nerves and skeletal muscles in patients and transgenic rodents with ALS [160]. In contrast to macrophages for which existing evidence on their potential beneficial or detrimental role in ALS is conflicting [157], MCs have recently been demonstrated to be located in degenerating motor nerve endplates in SOD1G93A mice, and the degree of MC degranulation was shown to correspond to clinical disease endpoints [160]. Masitinib administration was shown to diminish NMJ denervation and motor impairment in SOD1G93A mice [160], thus providing evidence of the role of MCs in ALS. The exact mechanism is unknown; however, MCs have been hypothesized to enhance vascular permeability in the context of neuroinflammation, thus triggering further neutrophil recruitment, clustering and activation [157,161,162,163]. Kovacs et al. [164] verified the presence of MCs and many c-Kit+ progenitors in the motor neuron-vascular niche, thus further supporting MC pathogenic potential in the context of ALS. 

### 7.4. Mast Cells (MCs) in Huntington’s Disease (HD)

HD is an inherited autosomal dominant neurodegenerative disease. HD is caused by, to various degrees, amplification of a CAG triplet in the HTT gene, thus resulting in the production of a mutant Huntingtin protein (mHTT). Excitatory neurotoxicity is caused by mHTT in the striatum and cortex, linked to movement disorder with chorea and/or dystonia accompanied by cognitive, and neuropsychiatric symptoms [195]. Reactive astrocytes have been reported in pre-clinical stages of HD and their presence further correlate with later disease severity [165]. Moreover, reactive microglia have been reported in the striatum and cortex of HD, accompanying neuron loss, in postmortem brains [167]. Although a neuroinflammatory component has been identified in HD pathogenesis, the relative contribution of MCs in HD pathology remains to be elucidated. MCs express TLR-4, a receptor recognizing PAMPs and DAMPs, such as the Gram-negative bacteria-derived lipopolysaccharide (LPS) [166]. TLR-4 activation in MCs results in TNF production, thus promoting mouse survival following systemic administration of LPS [166]. Perez-Rodriguez et al. [166] discovered that mHTT promotes TLR-4 receptor internalization, an important step in the signaling axis that affects MC activation. Thus, mHTT expression inhibits TLR4-dependent signaling, resulting in impaired innate immune responses and altering MC-dependent inflammatory response in vivo [166].

### 7.5. Mast Cells (MCs) in Multiple Sclerosis (MS)

MS is a multifactorial chronic demyelinating disorder of the CNS that displays inflammatory and neurodegenerative origins. It affects approximately 2.9 million people worldwide and is considered to be the most common cause of neurologic disability in young adults [196]. MS is frequently diagnosed between the ages of 20 and 50 years, with females experiencing it more often than males [197]; nevertheless, cases of pediatric and late onset MS are not rare [198]. In Greece, it is estimated that approximately 21,000 people are affected by MS [199]. 

MS has traditionally been regarded as a disease primarily of neuroinflammatory origin, with secondary neurodegeneration occurring later in the disease course. In MS, auto-activated T cell recognizing myelin antigens infiltrate the CNS, thus causing multi-focal demyelination in the context of neuroinflammation [200]. The majority of MS cases are diagnosed with the relapsing–remitting form of MS, a disease form with evident disease activity due to neuroinflammation. However, following several years, these patients are expected to develop secondary progressive disease, as neurodegeneration becomes increasingly evident as a result of accumulating age in combination with long-term disease pathology [201,202]. Of note, biomarkers that depict disease pathology and thus exhibiting potential to facilitate disease management and/or to estimate prognosis are currently lacking and are the focus of extensive research [203,204,205]. According to the latest international classification, there are three main types of MS: relapsing-remitting MS (RRMS), characterized by sub-acute episodes of neurological disability and intermittent periods of remission; primary progressive MS (PPMS), characterized by gradual accumulation of neurological disability from the disease onset; and SPMS, characterized by gradual accumulation of neurological disability following to the RRMS form [206]. The diversity in the pathophysiology of the two latter courses is still uncertain. The underlying mechanisms of MS are yet to be clarified [207,208], and the management relies on immune-modifying agents [204,209,210]. About 50% of individuals with MS display impairments in a wide variety of cognitive domains; among them, deficits in cognitive processing speed and working memory are the most frequently observed [211,212]. Additional cognitive domains that appear to be regularly affected by MS are diverse other aspects of memory, attention and executive functions [213,214]. Cortical inflammatory process, neurodegeneration, demyelination, axonal damage, axonal loss, oligodendrocytes, mitochondrial disfunction, microglia stimulation, oxidative and nitrosative stress are underlying processes of MS [200,215]. Myelinated axons in the CNS are the target of MS attacks, which can induce varying degrees of damage to both myelin and axon [216]. Reactive astrocytes sustained multifaceted contribution to neuroinflammatory outcomes and oligodendrocyte and neuronal function through chronicity, imply that they could be an integral cell type that can govern the pathophysiology of MS [217]. 

Beyond symptoms directly related to MS, typical comorbid conditions often diagnosed in people with MS include hyperlipidemia, hypertension, GI diseases, chronic lung disease, thyroid disease, obesity and neuropsychiatric disorders [218,219,220]. Comorbid conditions impact the disease course and affect treatment selection, adherence and outcome. In this sense, several intertwined issues emerge concerning diagnostic delay, degree of disability progression and additive effect on mortality [221]. Early diagnosis of comorbid conditions in clinical practice is of great importance to prevent a negative effect on MS [220]. However, little is recognized regarding the mechanisms of neurodegeneration in this disorder, a limitation that declines the development of more effective managements for MS [222].

Recently, the neurodegenerative component of MS has been the focus of extensive research, as it has been acknowledged that neurodegeneration contributes to the disease pathology across the disease course [200]. Moreover, although the activation of the adaptive immune system has been thoroughly interrogated in MS, the relative contribution of innate immune responses, especially in association to the chronic, more subjective to the role of neurodegeneration, disease stages, remains less elucidated [168]. MCs have been reported to contribute to MS pathogenesis by mediating inflammation and demyelination, acting as antigen-presenting cells towards T cells and/or by disrupting the BBB (reviewed in [169] and in [147]). With respect to the CNS MCs, MC inflammasome was shown to play a pivotal role in the meningeal inflammation that, at least in part, determines disease severity in a rodent model of MS [171]. The development of EAE reportedly involves MCs accumulation in the CNS, as shown in rats and mice [172,173]. What is more, evidence suggest that perivascular MCs may pose potential to regulate BBB permeability, an aspect important for MS pathogenesis [170].

Activated MCs appear to be involved in both innate and specific immunity, and their contribution to the diverse stages of MS and the development of EAE, the major animal model of MS, is significant [50,223]. MCs inhabitant in the dura mater and pia mater also aggravate EAE, through inducing CNS inflammatory cell influx [163]. According to Christy and Brown (2007), the most detrimental effects of MC occurrence in the CNS through MS/EAE development include the BBB permeability and a subsequent increase in the number of cells that access the CNS, which is mediated by histamine, chemokines, and leukotrienes. MCs could also contribute to epitope spreading through tissue damage provoked by proteases, T helper (Th) 1 and Th17 differentiation by releasing polarizing cytokines (IL-12 for Th1 and IL-6 plus TGF-β for Th17), and a reduction in the number of Tr1 regulatory cells by a direct interaction involving the OX40L/OX40 pair [163]. The contribution of MCs in MS is shown by gene overexpression of chemical mediators and inflammatory cytokines. MCs mediate inflammatory process and demyelization through presenting myelin antigens to T cells and/or disrupting the BBB, thereby allowing entry of inflammatory cells and cytokines in the brain, leading to MS neuropathy [169]. In the CNS, MCs are critically positioned in the perivascular area and produce numerous pro-inflammatory and vasoactive molecules that can disturb the BBB, an effect antecedent any clinical or pathological marks of MS [224]. In MS, brain MCs seem to be activated by neural agents, such as substance P, myelin basic protein, the principal MS antigen, and corticotropin-releasing hormone, caused by acute stress, which induce release of several inflammatory mediators involved in the pathophysiology of MS [224]. MCs are frequently also located near to MS plaques, and the myelin basic protein can trigger human cultured MCs to induce IL-8, TNF-α, tryptase, and histamine, contributing to the pathophysiology of MS. MCs might provide T cell activation because addition of MCs to anti-CD3/anti-CD28 activated T cells rises T cell activation above 30-fold, leading to MS neuropathy [225]. Therefore, the option to employ MC-targeted drugs to control this disease appears to be a promising therapeutic strategy. In this respect, for instance, pretreatment with the flavone luteolin completely inhibit MCs s and T cell activations, thus constitute a new therapeutic option for MS [225]. Moreover, ketotifen fumarate, a second-generation antihistamine agent, inhibits MCs exocytosis among other effects. Its early intervention seems to be very effective in controlling both the prevalence and severity of MS [226].

## 8. Impact of Gut–Brain Axis (GBA) and the Activation of Mast Cells (MCs) on Neurodegeneration: A Potential Role for *Hp* Infection

The GBA represents an important regulator of the brain’s immune responses with implication for neuroinflammation, thus bearing potential as a therapeutic target against the development and progression of neurodegenerative diseases [227]. The gut microbiome provides stimuli to the CNS innate immune cells, namely, the microglia, as well as to astrocytes and oligodendrocytes, via metabolites, gut microbiota-related neurotransmitters and gut hormones [228,229,230,231]. Growing evidence elucidates aspects of the GBA subjected to potential modifications, such as the intestinal barrier [232], the BBB [233], the meninges [234] and the peripheral immune system [235], as measures to ameliorate neuroinflammation in the context of neurodegeneration. Moreover, pre-clinical and clinical evidence on the beneficial application of probiotics [236], prebiotics [237] and fecal microbiota transplantation [238] in neurodegenerative diseases further underline the potential of the GBA to affect brain pathology. MCs are mediators of stress and neuroinflammation, as they have been shown to release CRH with systemic effects [239]. In the context of the GBA, MCs have been advocated as a cellular population with immune-modulatory potential [32], as MCs are abundant in the gut and present, to a lesser degree, in the brain upon neuroinflammation, shown to be capable of interacting with resident innate immune cells of the CNS, namely, the microglia [240]. Moreover, intestinal MCs have been shown to participate neuronal and endocrine signaling, thereby modulating neuroinflammation in the context of neurodegeneration [241]. Whereas MCs have been advocated to participate in neuroimmune signaling in the GBA [242], thorough mechanistic insight regarding the underlying mechanisms of this interaction is currently lacking.

An association between *Hp* infection and neurodegenerative diseases has been previously well described. MetS may be one of several common denominators in this association. MetS has been related to several brain neurodegenerative disorders. For instance, patients with HD exhibit peripheral IR, and a high risk of T2DM. Cellular cholesterol metabolism may be disrupted in HD, and it is advocated that the mHTT protein impacts cholesterol transport within the cell. Furthermore, patients with HD exhibit cerebral cortical hypoperfusion, even prior to the manifestation of cognitive symptoms and increased regional BBB permeability in the caudate nucleus, similarly to AD and PD [27,243].

IR is considered as a key factor contributing to MetS-linked pathology, in addition to well-described genetic and environmental factors [244]. In the context of MetS, glucose, lipid, and protein metabolism are impaired and ultimately lead in IR, obesity and CVD [245]. Growing evidence links *Hp* infection and IR/MetS, as well as T2DM, dyslipidemia, hypertension, MASLD and neurodegenerative and cardio-cerebrovascular diseases [69,246,247,248]. Underlying mechanisms by which MetS may be associated with brain pathology include for instance, but not exclusively, the disruption of the BBB, the triggering of neuroinflammation, the disruption of amyloid clearance and of the homeostasis of the cerebral blood vessels, thus resulting reduced cerebral blood flow, brain microenvironment alterations and disruption of the neurovascular unit [249,250,251,252]. *Hp*, as mentioned, is a common pathogen with its global prevalence approximating 58% [253]. *Hp* infection is associated with MetS-related systemic pathologies, including cardio-cerebrovascular and neurodegenerative disorders [86,87,115,128,246]. Moreover, *Hp* bacterial virulent factors may activate MC migration and the production of pro-inflammatory cytokines in the context of innate immunity. *Hp*-neutrophil-activating protein has been shown to induce degranulation of stored chemical mediators from MCs [254]. Oral administration of the *Hp*-related VacA in mice causes MC accumulation in the gastric mucosa [255] and the density of MC has been advocated as means of evaluating gastritis activity, in terms of histopathology, in patients with *Hp* infection [256]. However, the exact implications of MC–gastric epithelial cell interaction upon *Hp* infection in humans remain to be elucidated [257]. Recently, gastric IL-33 was found to be increased in the gastric epithelium of patients and mice with *Hp* infection, a result associated with the degree of bacterial expansion and the severity of gastritis [258]. Gastric IL-33 resulted in increased TNF-α production from MCs in vitro, and in vivo and inhibited gastric epithelial cell proliferation, thus further promoting *Hp*-associated gastritis and bacteria proliferation. The study provided evidence of *Hp*, gastric epithelial cells, IL-33, MCs, and TNF-α forming a complex regulatory network that, when disrupted, contributes to *Hp* infection-associated gastritis [258]. Eosinophilic esophagitis (EoE), an allergen/immune-mediated disease, also provides a model for MC association with *Hp* infection-related pathology. Recent evidence suggests a relatively protective effect of *Hp* infection in EoE, an observation that was not, however, further verified [259]. Although EoE is characterized by allergic reaction, thus typically Th2 immune response, a subset of older patients who exhibit intense allergic reaction at onset, also show activation of Th1 and Th17 pro-inflammatory responses [260], as in the case of *Hp* infection [261,262,263]. In the context of EoE, MCs mediate inflammation and fibrosis via the production of TGF-β and tryptase, thus further inducing smooth muscle contraction, esophageal spasm and collagen secretion [264,265]. In addition, TGF-β [266] and TGF-β-inducing protein from *Hp* mediate the pathogenesis of *Hp* infection [267]. *Hp* cytotoxin VacA induces the production of pro-inflammatory cytokines, such as TNF-α from the mentioned BMD-MCs, a cytokine reportedly overexpressed in esophageal epithelial cells in the context of EoE [268,269]. TNF-α stimulates MCs [270,271], a mechanism linked with *Hp* infection -associated chronic urticaria [272]. Interestingly, EoE-related activated MCs, beyond achalasia, have been implicated in the pathogenesis of neurodegenerative disorders such as AD and PD [273]. In this respect, *Hp* infection may contribute to the pathophysiology of achalasia, which, apart from AD and PD, is also associated with Guillain-Barré syndrome, by inducing autoimmunity and apoptosis. Thus, *Hp* eradication may offer benefit through amelioration the apoptotic loss of the esophageal wall ganglion cells and their axons [274].

MC activation in the context of *Hp* infection and the related MetS may contribute to the induction of CNS pathology via several mechanisms: *Hp*-induced MCs alter the balance of the GBA. As indicated by several CNS pathologies, such as infection, trauma and/or stress [42], MCs are contributors in the communication between the CNS and the systemic circulation, an observation with implication for neuroinflammation and neurodegeneration. For instance, as *Hp*-associated VacA shifts BMD-MCs towards the production of including TNF-α and other pro-inflammatory cytokines [275] and BMD-MCs are in proximity to the components of BBB, MCs may alter BBB permeability by affecting the microenvironment in terms of produced cytokines and chemokines. Moreover, *Hp* induces, via stress and/or increased gastrin production, local and systemic activation of MCs with the expression of VEGF, IL-8, chymase or tryptase, thus further disrupting the BBB and promoting immune cell infiltration towards the CNS. An additional mechanism is the activation of microglia in response to production of tryptase and histamine by MCs. Microglia secrete cytokines and/or chemokines into the microenvironment and further recruit MCs, eosinophils, monocytes and neutrophils, thus exacerbating neuroinflammation [276,277]. *Hp* and the associated activation of MCs in the context of *Hp* infection are, therefore, potential contributors of pro-inflammatory processes. MCs, in the presence of activated microglia, exhibit upregulated expression of TLR-2 and -4, both also expressed by *Hp*, thus rendered more responsive to endotoxin stimuli and microbial LPS [278]. 

These observations underline the complex interaction and the potential of MCs, in the context of *Hp* infection and in relation to microglia and other immune cells in the CNS, to alter the tissue microenvironment and to contribute to several neurodegenerative pathologies. In view of the recent recognition that neuroinflammatory processes have received in the context of diseases previously regarded as “primary neurodegenerative”, MCs and *Hp* infection may offer targets of intervention with potential therapeutic implications. More clinical and translational evidence is needed to elucidate in depth this field. 

## 9. Conclusions

In conclusion, this review underscores the complex interplay between *Hp* infection, MCs activation, and neurodegenerative diseases, particularly within the context of MetS. The findings suggest that *Hp* may play a significant role in promoting neuroinflammation and neurodegeneration through mechanisms such as IR, compromised BBB integrity, and altered immune responses. MCs emerge as key mediators in these processes, as their activation can lead to the release of pro-inflammatory cytokines and contribute to an unfavorable microenvironment for neuronal health. Importantly, the evidence linking *Hp* infection with MetS highlights a critical area for further exploration. Future research should focus on elucidating the specific molecular pathways that connect *Hp* to neurodegenerative pathologies, examining the potential therapeutic benefits of MCs modulation in neuroprotection, and investigating the bidirectional influences between gut health and brain function. Additionally, clinical studies are warranted to evaluate the impact of *Hp* eradication on neurodegenerative risk in at-risk populations, providing insights into the GBA’s role in overall neurological health and opening avenues for novel preventative and therapeutic strategies. This multifaceted approach could significantly advance our understanding of neurodegeneration and foster the development of targeted interventions to mitigate its progression.

## Figures and Tables

**Table 1:** Mast cells and mechanisms of neuroinflammation in neurodegenerative diseases.

Disease	Major Disease Mechanisms	Evidence of Neuroinflammation /Key Players	Evidence of MC Involvement
AD	oxidative stress [132]	IL-1, IL-6, IFNγ [133,134,135]	MCs recruited into amyloid plaques [133,134,135]
	hyperphosphorylation of tau protein [136]		phagocyted amyloid particles in MCs in skin and gastric samples—histamine release [137]
	Aβ accumulation [138]		SAA causes MCs degranulation, cytokine release (TNF-α and IL-1) and chemotaxis [137]
	impairment of cholinergic transmission [139]		MC granule material causes SAA degradation into proto-fibrillar intermediates [140].
			Aβ contributes to the degranulation of prefrontal cortical MCs, via the enhancement of Cx43 and Panx1 HC flux [137].
			MCs detect low-solubility amyloid particles and to migrate to plaque sites, where they trigger the release of inflammatory mediators and disrupt the BBB [137].
			MCs release MMP 2 and 9 and VEGF, leading to vascular leakage, CNS leukocyte infiltration, and edema [141].
			MCs detect IL-33 and act as early responders to brain injury via the release of pre-stored TNF [142] and VEGFs thus further recruiting leukocytes and MCs [143,144,145].
			MCs in stress-induced inflammation: release of CRH and further activation of MCs and microglia. Chronic stress triggers MC degranulation causing pro- inflammatory mediator release, synaptic loss, BBB disruption and neuroinflammation [146,147].
PD	loss of DA neurons in the SN and VTA [148]	Activated microglia and astrocytes present in areas with midbrain DA neuron loss [149]	Brain MCs neurotoxicity in the SN [150].
	reduced DA transmission to STR [148]	Accumulation of misfolded α-synuclein linked to dysregulated immune responses in the CNS [151].	Administration of MPTP after MC reconstitution in rats shown to increase oxidative stress and to alter levels of MDA, GSH, SOD and GPx [152].
	accumulation of α-synuclein [152]	PD-associated polymorphisms in the HLA-DR gene [151].	MCs are attracted and activated in the SN region of MPTP-induced mice: releasing inflammatory mediators (histamine, LTs, TNF-α, and IL-1, thus contributing to TH+ DA neuron loss in the SN [153]
	intracellular mitochondrial dysfunction-induced ROS generation [152]		Increased activated MCs in PD brains, particularly in the midbrain and the striatum and increased IL-33 expression linked to cell injury [146].
	dysfunction in the ubiquitin-proteasomal and autophagy- lysosomal system [152]		NLRP3 inflammasome’s involvement in Parkinson’s disease [132,154,155]. peripheral tissue MCs in CAPS patients express inflammasomes and produce IL-1 [156]—potential role for CNS MCs Limitation: CNS MC expression of the NLRP3 inflammasome has not been directly studied
ALS	degeneration of motor neurons [157]	Reactive immune cells in postmortem tissue of patients with familial and sporadic ALS [158,159].	Degranulating MCs present in the quadriceps muscle of patients with ALS [158,159]
	distal motor axonopathy [157]		MCs and neutrophils surrounding motor axons in SOD1G93A rats with ALS [158,159]
	impaired axonal transport [157]		Masitinib results in reduced infiltration by MCs and neutrophils, ameliorates axonal pathology and secondary demyelination, and hinders the loss of type 2B myofibers in SOD1G93A rats [158,159]
	mitochondrial function defect [157]		The degree of MC degranulation corresponds to clinical disease endpoints in SOD1G93A mice [160].
			MCs are regarded to enhance vascular permeability in the context of neuroinflammation, thus triggering further neutrophil recruitment, clustering and activation [157,161,162,163].
			Presence of MCs and many c-Kit+ progenitors in the motor neuron-vascular niche [164]
HD	production of a mHTT	Reactive astrocytes have been reported in pre-clinical stages of HD. Their presence correlates with later disease severity [165].	mHTT promotes TLR-4 receptor internalization, thus affecting MC activation [166]
	Excitatory neurotoxicity caused by mHTT in the striatum and cortex	Reactive microglia have been reported in the striatum and cortex of HD, accompanying neuron loss, in postmortem brains [167].	
MS	auto-reactive T cells recognizing CNS myelin antigens	Activation of the adaptive immune system [168]	MCs may act as antigen-presenting cells towards T cells [147,169]
	multi-focal demyelination	Microglia activation [168]	MCs disrupt the blood–brain barrier [170]
	neurodegeneration		MC inflammasome plays pivotal role in the meningeal inflammation in EAE [171].
			EAE development involves MC accumulation in the CNS [172,173].

Alzheimer’s disease (AD); amyloid beta (Aβ); interleukin-1 (IL-1); interleukin-6 (IL-6); interferon gamma(IFNγ); mast cells (MCs); amyloid A protein precursor serum amyloid A (SAA); tumor necrosis factor alpha (TNF-α); connexin-43 (Cx43); pannexin 1 (panx1); hemichannel (HC); blood–brain barrier (BBB); metalloproteinase 2 (MMP2); metalloproteinase 9 (MMP9); vascular-endothelial growth factor (VEGF); central nervous system (CNS); interleukin-33 (IL-33); corticotropin releasing hormone (CRH); Parkinson’s disease (PD); dopaminergic (DA) neurons; substantia nigra (SN); ventral tegmental area (VTA); striatum (STR); methyl-4-phenyl-1,2,3,6-tetrahydropyridine (MPTP); malondialdehyde (MDA); glutathione (GSH); superoxide dismutase (SOD); glutathione peroxidase (GPx); leukotrienes (LTs); human leukocyte antigen—DR isotype (HLA–DR); tyrosine hydroxylase (TH); dopaminergic (DA); reactive oxygen species (ROS); nucleotide-binding domain, leucine-rich-containing family, NOD-like receptor protein 3 (NLRP3); cryopyrin-associated periodic syndrome (CAPS); amyotrophic lateral sclerosis (ALS); superoxide dismutase (SOD); Huntington’s disease (HD); mutant Huntingtin protein (mHTT); toll-like receptor 4 (TLR-4); multiple sclerosis (MS); experimental autoimmune encephalomyelitis (EAE).

## Data Availability

No new data were created.
